# Hurdles to the Development of Effective HBV Immunotherapies and HCV Vaccines

**DOI:** 10.20411/pai.v2i1.201

**Published:** 2017-04-09

**Authors:** Almudena Torres-Cornejo, Georg M. Lauer

**Affiliations:** 1 Gastrointestinal Unit and Liver Center, Massachusetts General Hospital and Harvard Medical School, Boston, Massachusetts

**Keywords:** hepatitis-B, hepatitis-C, research funding, immune response, T cell, antibodies, vaccine, immunotherapy

## Abstract

Chronic infections with HBV and HCV continue to be major public health problems, with hundreds of millions of people infected worldwide; this is despite the availability of both an effective prophylactic HBV vaccine for more than 3 decades and potent direct antivirals for HBV and, more recently, HCV infection. Consequently, development of HBV immunotherapies and prophylactic HCV vaccines remains extremely urgent, but limited funding and significant gaps in our understanding of the correlates of immune protection pose serious hurdles for the development of novel immune-based interventions. Here we discuss immunological questions related to HBV and HCV, some shared and some pertinent to only 1 of the viruses, that should be addressed for the rational design of HBV immunotherapies and HCV vaccines.

## CHRONIC VIRAL HEPATITIS: A CONTINUED CHALLENGE TO GLOBAL HEALTH

Chronic viral hepatitis remains a vast global health problem. Around 360 million people worldwide are chronically infected with hepatitis B virus (HBV) and 130–150 million with hepatitis C (HCV) [1–3]. Together, these two infections are the cause of death for approximately 1.4 million people every year. Despite the availability of effective HBV vaccines and antiviral treatments, and the recent introduction of powerful direct-acting antivirals (DAAs) for HCV, we are not close to reasonably claiming “Mission Accomplished”, if our goals are to eliminate or even significantly diminish disease from these 2 hepatotropic infections in all parts of the world.

Three decades have passed since the first recombinant HBV vaccine was approved in the United States [4], and HBV vaccines have been shown to be safe and effective in > 90 percent of those vaccinated against all HBV serotypes and genotypes. The implementation of comprehensive HBV vaccination programs has now resulted in a significant decrease in the incidence and prevalence of HBV infection, but only in countries where the local resources enabled the establishment of these programs some decades ago [5]. In recent years we have seen further increases in the worldwide use of the HBV vaccine, with the WHO estimating in 2015 that 83% of all 1-year-olds had received a third dose of HBV vaccine. Nevertheless, substantial vaccination gaps exist, especially in many parts of Africa [6, 7]. Given the long delay between the establishment of effective vacci-nation programs and their impact on the prevalence of chronic HBV infection [8], the challenge of effectively treating millions of chronic hepatitis B patients will remain for the foreseeable future. Since current treatments rarely induce permanent viral control and thus need to be given in perpetuity, new therapeutic approaches leading to full viral eradication or at least functional cure are urgently needed.

For HCV infection, the situation is different, but equally challenging. No prophylactic vaccine is available, but the most recent DAA therapies are extremely effective and cure chronic infection in almost all treated patients [9]. The main challenge will be to make therapies affordable everywhere and to improve local medical infrastructure to allow for diagnosis and then treatment of most patients [10, 11]. The difficulties and delays in implementing universal HBV vaccination programs are a clear warning that such complex programs are not easily implemented on a global scale, and we should not expect the worldwide HCV epidemic to be dramatically curbed in the near future. In this situation, HCV vaccines remain an attractive complement to antiviral therapy, especially for areas with limited resources and high HCV prevalence. Additionally, in countries that have started to treat all diagnosed HCV patients, ongoing injection-drug use puts a significant subgroup of patients at risk for new and re-infection [12]. Many countries, including the United States, have seen a dramatic surge of intravenous drug use [13], most notably of heroin. Coincidentally, a wave of new HCV infections among young users has been observed in Massachusetts (1,026 reported cases from 2007–2009) [14] and other jurisdictions in the US [15–17] signaling a dramatic new HCV epidemic. Thus, an effective HCV vaccine remains highly desirable even in the era of DAA therapy.

**Figure 1:**
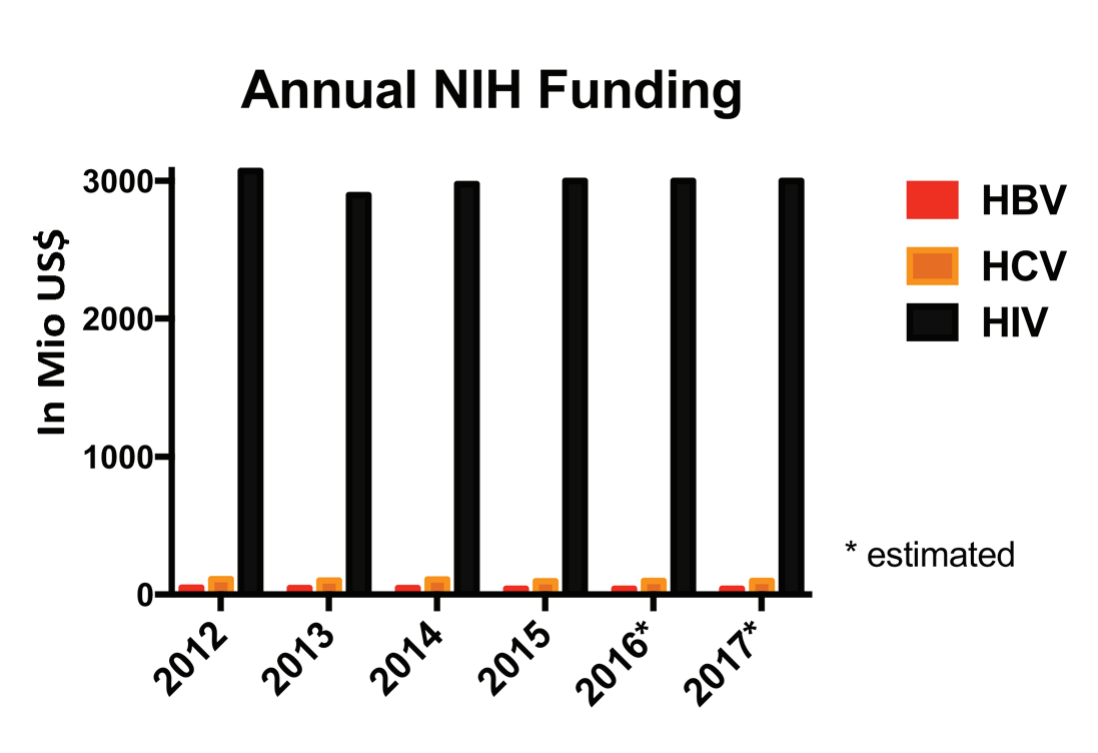
NIH research funding for HCV, HBV and HIV infection. Annual report of NIH funding, expressed as millions of dollars,(US) for research related to HIV (black bars), HCV (orange bars) and HBV (red bars) from 2012 to 2017.

Considering this information, it is evident that research efforts aiming to develop HBV immunotherapies and HCV vaccines should remain a high priority. Indeed, interest in HBV research has recently been invigorated, especially within the pharmaceutical industry. For many years, the availability of HBV vaccines and treatments, despite the evidence for continued high rates of HBV related morbidity and mortality around the globe, seems to have almost paralyzed funding agencies and industry. During those years, the annual HBV meeting was a small gathering of the most dedicated scientists, as public funding for HBV research remained flat or was even declining from already low levels [18]. Overall, combined NIH funding for HBV and HCV research is less than 5% of the annual expenditures for HIV (Figure 1). Because only a small fraction of these funds is spent on immunological studies, it is not surprising that the field is lacking the depth and breadth of investigation that would lead to faster progress and the consilience of findings from overlapping studies from different laboratories. Now, the same premature label of “disease of the past” has become attached to HCV infection. It is not uncommon to hear in NIH study sections that HCV research supposedly lacks relevance. At meetings and conferences, we regularly hear the question “what are you going to work on now?” [19]. We think the answer is obvious and we must continue studying the immunology of HBV and HCV until we have successful interventions that will truly diminish the dramatic impact that these 2 viral infections continue to have on millions of people worldwide.

In this review, we will focus on key questions related to the immunological control of HBV and HCV and outline the current available data. We will also highlight the gaps in our knowledge that we see as critical to be filled to spur the development of effective vaccines and immunotherapies. While some of the challenges and questions are the same for both viruses, key differences in their clinical and virological course, and in the nature of the required immunological interventions, lead to partially different foci of immunological investigation.

## HBV AND HCV: DIFFERENT COURSES OF INFECTION

HCV typically results in a straightforward and linear clinical, virological, and immunological course of infection that is quite distinct from the much more complicated situation in HBV infection. Once a person becomes infected with HCV, they either develop chronic viremia, or completely resolve the infection, usually within 6 months of exposure [20]. After this decisive phase, virus-host interactions remain relatively stable, and most patients progress on a comparatively linear scale clinically, although at different speeds. The individual trajectory can range from a completely stable scenario with total absence of liver disease, to the development of severe active hepatitis and fibrosis followed by cirrhosis in only a few years. This may occur, for example, with HIV coinfection [20] [21]. However, in most HCV cases, the timespan from infection to severe liver disease is measured in decades, not years. In comparison, HBV infection evolves in a much more complex and dynamic way throughout its entire course, and many of the associated key immunological and virological mechanisms are not completely understood. First, there is an important age factor; as HBV infections in newborns or at a young age mostly result in chronic infection, while almost all adults will control HBV spontaneously [22]. In contrast, age is much less of a determinant for the outcome of HCV infection and younger age at infection is actually associated with a more favorable outcome [23]. Second, it is not quite clear whether full eradication of HBV is possible as part of its natural history, or whether HBV covalently closed circular DNA (cccDNA) persists in all subjects indefinitely, even in resolved infections with hepatitis B surface antigen (HBsAg) clearance and HBsAg seroconversion. Finally, chronic HBV infection itself is a dynamic and non-linear process, with different stages characterized by different combinations of viral replication levels, antibody (and T cell) response features, and changes in liver disease activity [24]. Understanding to what degree the immune response is responsible for these distinctive features of HBV infection, or whether it only responds to them, will be critical for designing successful immunotherapeutic interventions.

## HBV: AGE AND IMMUNE RESPONSE

**Figure 2:**
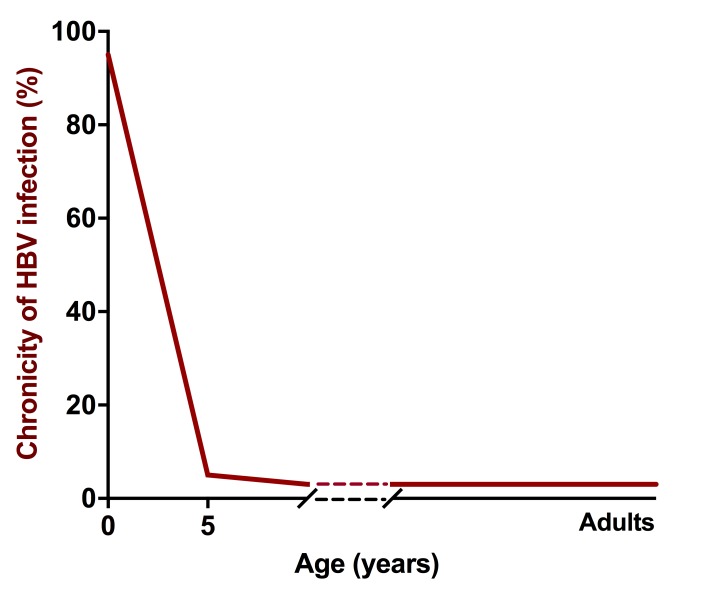
Risk of chronic Hepatitis B related to age. The risk of developing chronic HBV infection is strikingly age dependent.

The age-related difference in the clinical outcome of acute HBV infection is striking [25]. In adults, at least 95% of primary infections result in seroconversion to hepatitis B surface antibody (anti-HBs) and long-lasting immunity against reinfection [26]. In contrast, > 90% of newborns and 30% of children aged 1–5 years fail to resolve HBV and develop chronic infection [22, 27] (Figure 2). Chronic HBV infection in children is initially characterized by high viral loads (HBV-DNA) in the absence of clinical or biochemical signs of liver disease. Because HBV is not cytopathic by itself and the cellular immune response is thought to be the key mediator of liver inflammation in HBV infection, this has been interpreted as evidence that an immunotolerant state is key to HBV persistence, and maintained for many years post infection [28, 29]. However, at present our understanding of the early host virus interactions in newborns or young children exposed to HBV is still extremely limited, and therefore the exact gaps in the anti-HBV immune response that enable chronicity have not been well defined. The most specific hypothesis stems from a study in a mouse model of HBV infection, which suggests that maternal HBV e antigen induces PD-L1 expression and altered polarization in the macrophages of the offspring, leading to direct impairment of the HBV-specific CD8 response[30]. It remains to be seen how these findings translate to human HBV infection. Similarly, it remains unclear whether the subsequent “immunotolerant phase” is in fact characterized by an absence of adaptive immune responses or by the presence of immune responses that are unable to mediate both viral control and hepatic damage. In that context a recent study on CD8 T-cell responses had quite surprising results, suggesting that the weakness of inflammatory activity during the immunotolerant phase cannot be equated with the absence of HBV-specific T-cell responses [31]. The HBV-specific CD8 T-cell responses that were detected in children and young adults without signs of active liver disease unexpectedly displayed better functionality in terms of proliferation and cytokine secretion. Other studies were less successful in identifying T-cell responses in the immunotolerant phase [32, 33], so at this point it seems premature to close this chapter of investigation.

A comprehensive assessment of the global immune response against the development and evolution of HBV infection in children will be critical to define the determinants of protection as well as HBV viral persistence, and to better understand the immunological foundation from which later phases of chronic infection emerge. There are reasons for the sparseness of relevant data regarding childhood infection, for example, newborns identified to be at risk for infection can be successfully protected through concurrent active and passive immunization. In addition, only minimal amounts of blood can be drawn for analysis to investigate the sporadic infections in young children that still can be identified [34]. Nevertheless, newer technologies allowing comprehensive analysis of even single immune cells [35, 36] should allow us to finally decipher the immunological correlates of viral control, viral persistence, and the actual immune status during what we call the immunotolerant phase.

## WHAT IS “RESOLVED” ANTI-HBS POSITIVE HBV INFECTION? WHAT MEDIATES HBV RESOLUTION?

Clearance of HBsAg and the consecutive development of anti-HBs are the established hallmarks of HBV resolution [37]. This is regularly observed in adults with primary infection [38], but can also occur spontaneously on rare occasions, even after many years of chronic hepatitis B infection [24]. It is also the desired, though infrequent, outcome of current antiviral therapies [39]. However, what is referred to as resolved infection is not necessarily equivalent to the complete viral clearance observed in HCV infection. This is evidenced by small flares of HBV viremia that can sometimes be observed in people who have a positive test for anti-HBs [40]. In addition active HBV replication can recur during episodes of immune suppression, for example in patients undergoing ablative chemotherapies or immunomodulatory treatments with drugs like rituximab, although only in a small subgroup of patients [41, 42]. Underlying this phenomenon is the long-term persistence of cccDNA, the intracellular intermediate replication form of HBV [43]. The presence of cccDNA can only be reliably detected by analyzing liver tissue [44], which cannot routinely be sampled and is especially unlikely to be obtained from people without active liver disease. Therefore, we do not know if cccDNA persistence is universal in patients with resolved infection, or whether there are cases in which the immune response has truly eliminated all traces of HBV. This is a fundamental question to answer, because it will tell us whether immunothera-pies have the potential to achieve complete HBV eradication, or whether a functional cure is the most optimistic scenario for these therapies. If both scenarios do co-exist, there might be differences in the immune mechanisms mediating full clearance versus functional cure.

The fact that not only general immunosuppression but also specific immunomodulatory drugs, lead to loss of HBV control offers the opportunity to elucidate which immune mechanisms are the key mediators of HBV control. In particular, studies of the immunomodulators with more targeted functions might be instructive; however, their modes of action are more complex than is often appreciated. For example, rituximab is a monoclonal antibody targeting the CD20 antigen expressed on B cells, thus diminishing antibody production [45]. However, rituximab treatment also impairs CD4 T cells and alters CD8 T-cell distribution, which also could enable HBV recurrences [42, 46]. Nevertheless, analyzing the detailed effect of such drugs on virus-specific immune responses targeting HBV and other viruses will reveal important aspects of exactly how immunity is conferred.

While the exact contributions of many arms of the immune system in controlling HBV have not yet been defined, there is reasonably strong data that vigorous and multi-specific CD4 and CD8 T-cell responses are necessary. Studies of CD4 and CD8 T-cell depletion in chimpanzees have been shown to enable chronic infection [47, 48], while functional T-cell responses are associated with HBsAg clearance [49, 50]. Nevertheless, we need more data on the exact immunological requirements for achieving control in acute HBV infection and how subsequent long-term control is maintained. A recent study from Carlo Ferrari's group made a detailed analysis of HLA tetramer+ CD8 T cells from acute and chronic infection at the molecular level, and they found signifi-cant mitochondrial dysfunction in HBV-specific CD8 T cells during chronicity. This exemplifies the kind of immunological analysis that is now feasible and needed [51].

## THE IMMUNE RESPONSE IN NATURAL AND TREATMENT-ASSOCIATED TRANSITIONS BETWEEN DIFFERENT STAGES OF CHRONIC HBV INFECTION: DRIVER OR PASSENGER?

**Figure 3:**
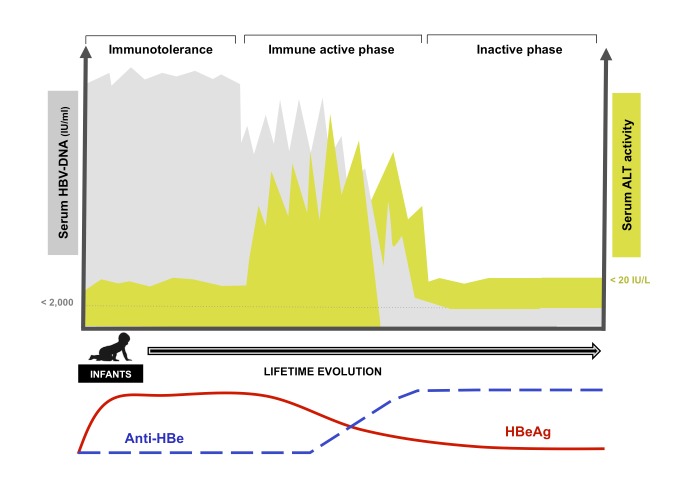
**Natural evolution of chronic HBV infection in infants.** The long-term course of chronic hepatitis B infection after infant exposure can be typically described in 3 different phases, characterized by different patterns of ALT elevation (green), HBV-DNA levels (grey), and the presence of HBe-Ag (red-line) or anti-HBe (blue).

Chronic HBV infection evolves in distinct stages that are broadly characterized by a combination of different levels of viral replication and liver disease activity, together with changes in HBV antibody profiles (Figure 3). The typical course of HBV disease after childhood infection, which often evolves over decades, ranges from the asymptomatic immunotolerant phase, with high viral replication and no significant liver inflammation and fibrosis, to the immune-reactive phase with lower viral replication but active inflammation, and finally to the inactive phase with low viral loads and no or minimal disease [52]. This later inactive phase can sometimes be followed by reactivation of disease activity in a variety of circumstances [53–55]. It should be noted, however, that this schematic sequential evolution of chronic HBV infection is not exactly replicated in every patient, as real-life infection, not surprisingly, comes in many shades of grey. Each of these distinct clinical phases and their transitions, and also those patients with unusual clinical features deviating from the classical course of HBV infection, have the potential to teach us about distinct modes of interaction between the virus and the immune system.

We have already discussed the immunotolerant phase; however, at the end of this phase clearly something remarkable must be happening, because after many years of uncontrolled infection patients spontaneously achieve viral control and also develop liver disease [56]. This seems to be a dynamic process, as significant fluctuations in both viral load and ALT can be observed in this period, until both viral load and liver-disease activity decline together with HBe seroconversion, during the transition to the inactive carrier state.

What initiates and drives these transitions? Thus far we have mostly circumstantial evidence. In some, but not all patients, ALT flares precede HBe seroconversion, potentially suggesting increased or functionally altered CD8 T-cell activity. Early puberty onset is associated with early seroconversion in both male and female children [57, 58], suggesting a hormonal trigger for the change in immunological control. Indicating a more specific immunological mechanism, certain HLA class I and class II alleles (HLA-B61 and HLA-DQB1*0503) [89–61], and also IL10 and IL12 polymorphisms associated with altered levels of circulating cytokines, have been linked to earlier HBeAg seroconversion in long-term studies from childhood to adult life [62, 63]. At this point we are a long way from identifying the exact immunological changes during the transitions from one clinical phase of HBV infection to another, and from understanding underlying causalities. This is also true for the occasional cases of spontaneous reactivation of active HBV-related liver disease in the absence of immunosuppressive therapies [64, 65]. Investigating these phenomena in humans is rather challenging, not least because of the slow and unpredictable evolution of HBV infection, and so this work does not fit well with the scientific funding cycle. The establishment of high quality, well-defined, and appropriately sampled clinical cohorts are the sound foundation of any high quality human research, but is labor and time intensive and rarely rewarded by scientific review panels. The resulting lack of support for cohort building as the basis for translational bench research has hampered both HBV and HCV research, especially given the lack of suitable animal models for immunological studies related to these viruses.

Analyses of immunological changes during HBV therapy are the logical extension of studies defining the immune response during the natural history of HBV infection. Since immuno-therapies will almost certainly not be fully effective on their own, they will most likely be given in conjunction with antiviral treatments to reduce the viral burden, because at least some viral escape mechanisms, such as T-cell exhaustion, are the direct consequence of antigen load [66]. Indeed, increases in functionality and frequency of HBV-specific CD4 and CD8 T cells have been observed during anti-HBV therapy [67, 68], although the improved responses are not effective enough to maintain viral control after cessation of therapy in most cases [39]. One obvious strategy in this context would be the addition of therapies restoring T-cell activity by blockade of T-cell inhibitory pathways, such as the PD-1/PD-L1 axis. This approach has been shown to be effective for resuscitating T-cell responses targeting human cancer [69, 70], and also to some degree HCV infection [71]. Many other T-cell inhibitory pathways have been discovered over the last decade, mostly in animal models of cancer and infection, and often their blockade had not only additive but also synergistic results for T-cell recovery [72–75]. On the other hand, recent data indicate that sometimes T cells that have been exhausted by inhibitory pathways for a very long time, such as in most cases of chronic HBV infection, might have epigenetic alterations, which could severely limit the potential for immune reconstitution by these mechanisms [76, 77]. Therefore, we should continue to explore additional, HBV-specific immune inhibitory mechanisms that could be equally or better suited for HBV immunotherapies. In addition, we need to consider that for HBV infection there is always the concern that strong invigoration of the immune response might have undesirable consequences, most notably ALT flares or even severe liver damage [22]. This underscores the primary need for understanding the immunological mechanisms of viral control and liver damage during the natural history of HBV infection, followed by studies on how anti-viral therapy could shift the balance between host and virus towards a situation more suitable to immunological interventions during the different phases of the infection. Only with this knowledge will it be feasible to rationally design immunotherapeutic interventions that can effectively push the immune response towards a profile associated with viral control and not liver injury, an immune profile that we know exists in the many individuals that have achieved sustained viral resolution.

## HCV INFECTION

HCV infection is unique among chronic viral infections in humans because a significant proportion of infected people, estimated to be 20% to 30%, can fully clear viremia spontaneously [20]. Typically, HCV clearance is achieved only within 6 months of infection, and after this spontaneous clearance, people who are later re-exposed to HCV, have a high likelihood of terminating viremia again [78, 79]. These observations support the idea that at least protection from chronic infection is highly plausible for HCV infection. However, we do not know if sterilizing immunity can be achieved by vaccination, nor what kind of vaccine-induced immune response would be required to assure protection from chronicity.

## CAN HCV VIRAL DIVERSITY BE OVERCOME?

**Figure 4:**
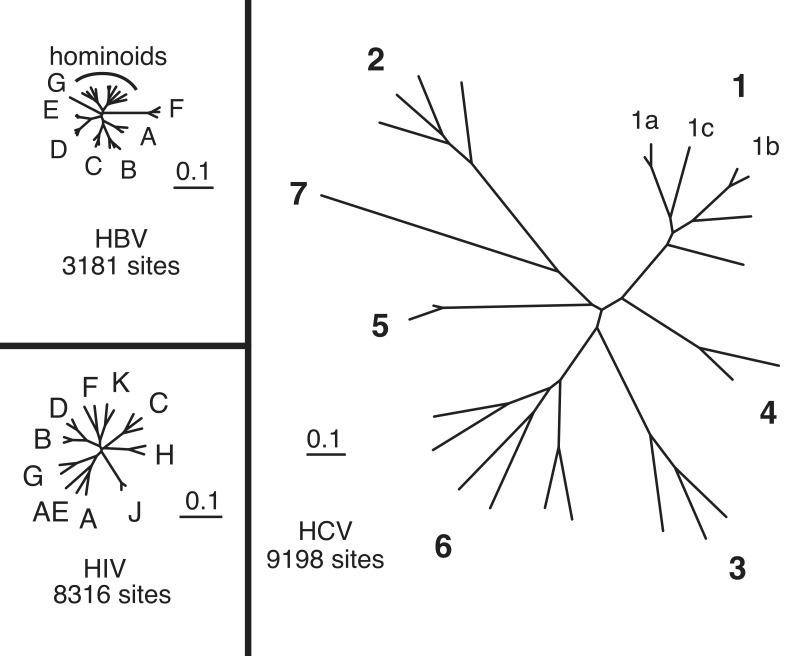
**Viral diversity of three viruses: HIV, HBV amnd HCV.** Phylogenetic trees for hepatitis B virus (HBV), human immunodeficiency virus (HIV), and hepatitis C virus (HCV) shown to the same scale in terms of nucleotide genetic distance, based on full-length genome nucleotide sequences. Sequences of representative strains for major genotypes were obtained from GenBank and aligned using ClustalX with minor manual adjustment, and then sites containing gaps were removed, resulting in an alignment of 3181 sites for HBV, 8316 sites for HIV, and 9198 sites for HCV. Maximum likelihood trees were inferred using PAUP* version 4b10, with the model (GTR+I+G in all three cases) and parameters selected by ModelTest 3.7 using the AIC criterion. Reproduced with friendly permission from Dr. Stuart Ray. (Figure 156-4 from Ray SC and Thomas DL. In: Bennett JE, Dolin R, and Blaser MJ (eds). *Man-dell, Douglas, and Bennett's Principles and Practices of Infectious Diseases*. 8th ed (updated). Churchill Livingstone Elsevier; 2015:1904-1927).

The imposing diversity of circulating HCV strains is the most challenging hurdle for effective vaccination. The HCV is a single-stranded, positive sense, RNA virus of approximately 9,600 nucleotides in a coding region that contains 1 large open reading frame flanked by non-translated regions at the 5′ and 3′ ends [80]. Due to the lack of a proof-reading function, its RNA-polymerase has a high error rate, estimated at 10-^4^ substitutions per site and round of replication, among the highest for RNA viruses including retroviruses [81]. Together with its high replication rate (approximately 10^12^ virions per day) [82] and the selective pressure exerted by the host immune system, this leads to the constant emergence of new HCV variants. The diversity starts within the host, as HCV infection is characterized by the co-existence of a swarm of closely related viruses, called viral quasispecies, at any given time. At the population level, viral diversity is much greater than that of other variable viruses, such as HIV and HBV (Figure 4), which is the result of HCV not only being highly variable but also having been present in humans for a very long time. We can now differentiate at least 7 different HCV genotypes and many more subtypes, with genetic variability between genotypes ranging from 31%–33% on the nucleotide level [83]. The genotypes of HCV show regional differences, examples being genotype 1 and more recently genotype 3, most frequently found in the US and Europe and genotype 4 being dominant in Egypt [80, 84]. This heterogeneity of infecting viruses makes development of a universal HCV vaccine extremely challenging. Even genotype specific vaccines would not be trivial to design, considering that the observed diversity even within HCV subtypes is as high as that of all the clades of HIV-1 group M. One suggestion to overcome this obstacle has been to use consensus sequences as immunogens to avoid inclusion of sequences that are already adapted to escape human immune responses, which would be the case for HCV strains isolated from infected patients [85, 86]. Another possibility is the use of mosaic sequences that can induce responses against multiple viral variants [87, 88]. There is an ongoing clinical trial using an HCV T-cell vaccine based on the complete non-structural region of a patient-derived genotype 1b sequence. The participants are injection drug users and are at high risk for HCV infection [89]; this study should reveal important insights into the impact of HCV variability on vaccine efficacy.

## IS STERILIZING IMMUNITY POSSIBLE?

Antibody mediated sterilizing immunity is the classic mechanism of protection used by traditional antiviral vaccines [90, 91]. However, it remains an open question whether sterilizing immunity is a feasible goal for HCV vaccines. Humans or chimpanzees, who have previously resolved HCV infection spontaneously, produce neutralizing antibodies, yet reinfection even with closely related viral strains can be observed. On the other hand, it has been reported that some chimpanzees were at least protected from a second homologous challenge [92, 93], indicating that failure of protection is not necessarily caused by an inability to prevent infection through neutralization, but rather by the high viral diversity of HCV as already described. This is not surprising, since the viral envelope regions most accessible for neutralization also contain some of the most variable regions of HCV [94, 95], as a direct consequence of antibody-mediated immune pressure. Nevertheless, several highly cross-reactive antibodies targeting highly conserved HCV epitopes have been identified [96], and together with antibody modifications overcoming neutralization resistance their potential in a prophylactic vaccine conferring sterilizing immunity should be explored.

## WHAT KIND OF IMMUNE RESPONSE WILL RELIABLY PROTECT FROM CHRONIC INFECTION?

Given the uncertain potential for a vaccine inducing sterilizing immunity, a second line of defense should be part of the strategy to protect against chronic hepatitis C. The dichotomy of natural HCV outcome, with 20% to 30% of infected people clearing viremia spontaneously [20], is proof that HCV can be immunologically controlled after infection. The potential for a vaccine to at least provide protection from chronic infection is further underscored by the fact that reinfection in people and chimpanzees who have previously cleared HCV is self-limited in most cases [78], with lower peak viremia and more rapid viral clearance. However, we do not know what kind of immune response is essential for viral control and what kind of vaccine-induced memory response would ensure protection on exposure.

With regard to protective immunity in established HCV infection, the most convincing data support an absolute requirement for HCV-specific CD4 as well as CD8 T-cell responses. Depletion of either cell type was associated with persistent infection in the chimpanzee model of HCV [97]. In addition, the emergence of HCV-specific CD8 T cells coincides with signs of viral control in both humans and chimpanzees [98, 99], whereas early defects in the proliferative capacity of HCV-specific CD4 T cells are the universally accepted hallmark of viral persistence [100–102]. It is much less obvious what quality or repertoire of T-cell response needs to be induced. One often reads that breadth and magnitude of the responses are critical, but in our opinion this is not fully supported by the experimental data. There is no doubt that with established HCV clearance or persistence, we observe much weaker and usually more narrow T-cell responses in patients with chronic infection. Nevertheless, studies at early time points in acute infection support the hypothesis that broadly directed and vigorous CD4 and CD8 T-cell responses are primed in most patients, and that maintenance of the responses is the most critical difference between self-limited and chronic infection [103–105]. Differences in viral control also manifest in the expression of inhibitory receptors such as PD-1 on T cells [106, 107] and other viral escape mechanisms, such as the occurrence of viral escape variants [85]. However, it is not clear whether these differences are the cause or consequence of differences in viral control, and it seems quite possible that additional early events initiate the distinct fate of the T-cell response in different outcomes of infection.

Very few studies of acute hepatitis C actually investigate the early immune response, before viral control or viral persistence are established. This is not surprising, given the usually unspecific symptoms of acute infection and the organizational challenges to identify and recruit subjects immediately after infection. Nevertheless, such studies are critical for understanding not only the consequences of distinct virological outcomes but also the drivers of immune control and failure.

Despite significant gaps in understanding of the immune determinants of HCV control, it seems a reasonable assumption that induction of a broadly directed response by both CD4 and CD8 T cells will be beneficial, if only because it will allow some redundancy when confronted with viral strains containing T-cell epitope variants. Careful analysis of ongoing vaccine trials should answer the question of whether T cells with a specific transcriptional profile and different cell subpopulations, especially T helper cells, are critical for protection, or whether a more rapid response time of any vaccine-induced memory T cells provides a sufficient advantage for the host. Additional valuable insights will come from analyzing patients that experience reinfection after previous clearance of HCV.

Antibodies specific for HCV might also contribute to HCV control after infection has been established, but the possible extent of their contribution has not been well defined and remains somewhat controversial. Clearly, HCV-specific antibodies do not seem an absolute requirement for spontaneous clearance of HCV, as self-limited infections have been observed in patients with antibody deficiencies, but they could still contribute to viral control after infection. Indeed, with the more recent introduction of neutralizing antibody assays for HCV we have seen good evidence suggesting a protective role for neutralizing antibodies in at least a subgroup of patients [91, 108]. In addition, non-neutralizing antibodies might contribute to viral control through virus-specific mediation of antibody-dependent cell-mediated cytotoxicity (ADCC) and other innate immune functions [109]. It seems likely that there is more than one immunological path to HCV clearance. If a vaccine can induce many different potential mediators of HCV control, it will be much for difficult for the virus to escape immune surveillance.

## HBV, HCV, AND THE LIVER

Understanding how immunity unfolds and is regulated within the liver as the site of infection will be critical for developing HBV immunotherapies as well as HCV vaccines. It has been well established from animal models, that the immunological environment of different tissues has a major impact on the immune response at the site of infection. In addition, the liver is a unique immunological organ, for both its tissue architecture and its role as the entry gate for molecules taken up in the gut [110]. Early studies on both HBV and HCV immunology often had to rely on liver biopsies, because immunological assays at the time were only capable of detecting specific T cells from the liver where they are present in much higher frequencies. Subsequently, more sensitive techniques have allowed analysis of virus-specific T cells from the blood, allowing for studies in many more subjects and at multiple time points. Despite this unquestionable improvement, some important dimensions of the immune response against hepatotropic viruses were also lost. Several groups continued to investigate intrahepatic T cells, resulting in important insights [111, 112], but only recently have we obtained the necessary tools to comprehensively study very small numbers of cells, even single cells [113, 114], and in great detail at the same time. While clinical biopsies have become much less important and frequent in both HBV and HCV infection, the ability to analyze very small populations of cells has enabled the use of fine-needle aspirates of the liver, which can be performed serially and outside the context of a clinically required biopsy [115]. We expect that this will lead to a completely new assessment of virus-host interactions directly at the site of infection. These kinds of studies should also allow an appreciation of intrahepatic interactions between different pathogens that can significantly alter the local environment, both viro-logically and immunologically. A prime example is the recent observation that successful therapy of chronic HCV infection using DAAs can be associated with the reactivation of previously controlled HBV [116, 117]. Whether this was caused by HCV eradication opening up replicative space in the hepatocyte population, or by changes in HCV-induced innate or adaptive immunity that had helped to control HBV, is not yet clear, but studies in such scenarios could contribute greatly to our understanding of complex immune surveillance networks at the site of infection.

## SUMMARY

The battle against both HBV and HCV infection would greatly benefit from having novel immunological weapons in its arsenal. In particular, we need therapeutic approaches mediating functional cure in HBV infection and prophylactic vaccines protecting from the development of chronic infection for HCV. In order to develop such interventions, we need to better understand the complex host and virus interactions in these viral infections and their relationship to viral control and viral persistence mechanisms. Some key questions overlap between the 2 viruses, most notably regarding the essence of what constitutes effective immunity and the characteristics of an immune response unfolding within the liver. However, each virus also poses a distinct set of questions related to their rather different clinical course and viral features, and because of the different nature of the desired immunological intervention. Recent technological breakthroughs have opened unprecedented opportunities for human immunology research, and with adequate funding scientific and medical breakthroughs should follow.
